# Three-Dimensional Helical Antenna Array with Circular Polarization

**DOI:** 10.3390/mi17091055

**Published:** 2026-09-03

**Authors:** Hui Peng, Kecheng Ye, Wei Nie, Yuxiang Li

**Affiliations:** 1 College of Electronic and Communication Engineering, Kashi University, Kashi 844000, China; 2 School of Communication and Information Engineering, Chongqing University of Posts and Telecommunications, Chongqing 400065, China

**Keywords:** circular polarization, planar helical antenna (PHA) array, substrate integrated waveguide (SIW), SIW transition

## Abstract

In this paper, a 4 × 4 planar helical antenna (PHA) array fed by a three-dimensional substrate integrated waveguide (SIW) power splitter is presented. The 4 × 4 array is composed of four 1 × 4 PHA subarrays, and each subarray is fed by the SIW power splitter. Since the helical antenna is end-fire, it could not be integrated into a 4 × 4 array in a 2-D plane. In order to realize a 4 × 4 array, a SIW twist vertical transition structure is designed to connect the subarray with the SIW power splitter. Finally, the proposed 4 × 4 helical antenna array is implemented and fabricated for measurement. The center frequency of the PHA is 11 GHz, the impedance bandwidth (IBW) is 10.16–11.99 GHz (16.64%), and the axial ratio bandwidth (ARBW) is 10.53–11.47 GHz with the peak gain of 18.44 dBi. The simulation and measurement results match each other well; therefore, the effectiveness of the proposed design is verified.

## 1. Introduction

Circular polarized (CP) antennas are widely utilized in satellite communications and radar systems due to their ability to mitigate multipath interference and polarization mismatch. CP implementation methods include patch antennas [1,2], cross dipoles [3,4,5,6], cavity antennas [7,8], horn antennas [9,10], spiral antennas [11,12], and helical antennas [13,14,15,16]. Among these, helical antennas stand out for their broadband operation, high gain, and axial radiation mode, which makes them ideal for long-range wireless applications. Additionally, substrate integrated waveguide (SIW) serves as an efficient feeding network for such antennas due to its low-loss characteristics.

However, the traditional helical antenna size is too large to meet the compact size requirement of modern wireless communication systems. Many designs have been made to miniaturize helical antennas. In [14], a helical antenna based on low-temperature co-fired ceramic technology is proposed. A coil of the antenna consists of a 1/3 turn open loop of three metal layers and a metal through hole, and its 4 × 4 array achieves a gain of 15 dBi at the center frequency, an impedance bandwidth (IBW) of 21.67%, and an axial ratio bandwidth (ARBW) of 20%. In [15], a planar double-arm helical antenna made of printed circuit board (PCB) is presented. The antenna is composed of metal strips and plated via holes on both sides. The single antenna achieves a gain of 7.8 dBi at its center frequency with an IBW of 54% and an axial ratio bandwidth of 34%. In [16], a single-arm planar helical antenna (PHA) manufactured by PCB technology is presented. The antenna also consists of two layers of metal strips and plated via holes, achieving a gain of 8 dBi with 54% IBW and 34% ARBW at the center frequency.

From the perspective of the manufacturing process, the PCB single-arm PHA reported in [16] offers significant advantages: First, based on a mature PCB process, it has the characteristics of a short fabrication cycle and low cost for mass production. Second, compared with the 20-layer low-temperature co-fired ceramic (LTCC) complex stacked structure in reference [14] and the two-arm symmetric design in reference [15], the single-arm topology greatly simplifies the structural complexity. However, this design has two major limitations: lateral dimensions limit the spacing of the elements, and coaxial feeding limits the ability of large-scale array integration. Recent studies have also investigated integrated circularly polarized arrays. In [17], the authors proposed a W-band 2 × 2 LTCC helical antenna array, while in [18,19] the authors developed 4 × 4 planar CP arrays with measured peak gains of 18.28 and 17.85 dBic, respectively. These works demonstrate the feasibility of high-gain CP array integration. However, most reported designs either rely on multilayer fabrication or employ broadside-radiating elements, while the two-dimensional integration of planar end-fire helical antennas remains challenging. A planar CP end-fire antenna array based on a slotted SIW was proposed in [20], while a low-profile SIW-based CP array using a single-layer PCB structure was developed in [21]. An X-band 4 × 4 CP array was presented in [22], and a differential-fed SIW cavity-backed CP array was investigated in [23]. These studies demonstrate the effectiveness of integrated feeding structures for CP array realization. However, the two-dimensional integration of planar end-fire helical antennas remains challenging.

In this paper, a 4 × 4 PHA array fed by an SIW feeding network is proposed. Firstly, the helical antenna unit is analyzed for further integration. Secondly, four helical antenna units are utilized to form a 1 × 4 PHA array fed by a 1-to-4 SIW power splitter. In order to unite four 1 × 4 PHA arrays to construct the 4 × 4 PHA array, a twist waveguide transition is designed to connect the SIW feeding network to the four 1 × 4 PHAs. The proposed antenna features good end-fire radiation performance and excellent circular-polarization characteristics while a compact size is obtained. The measurement results verify the design procedure.

## 2. Antenna Array Design

### 2.1. Antenna Unit Analysis and Design Procedure

**Figure 1:**
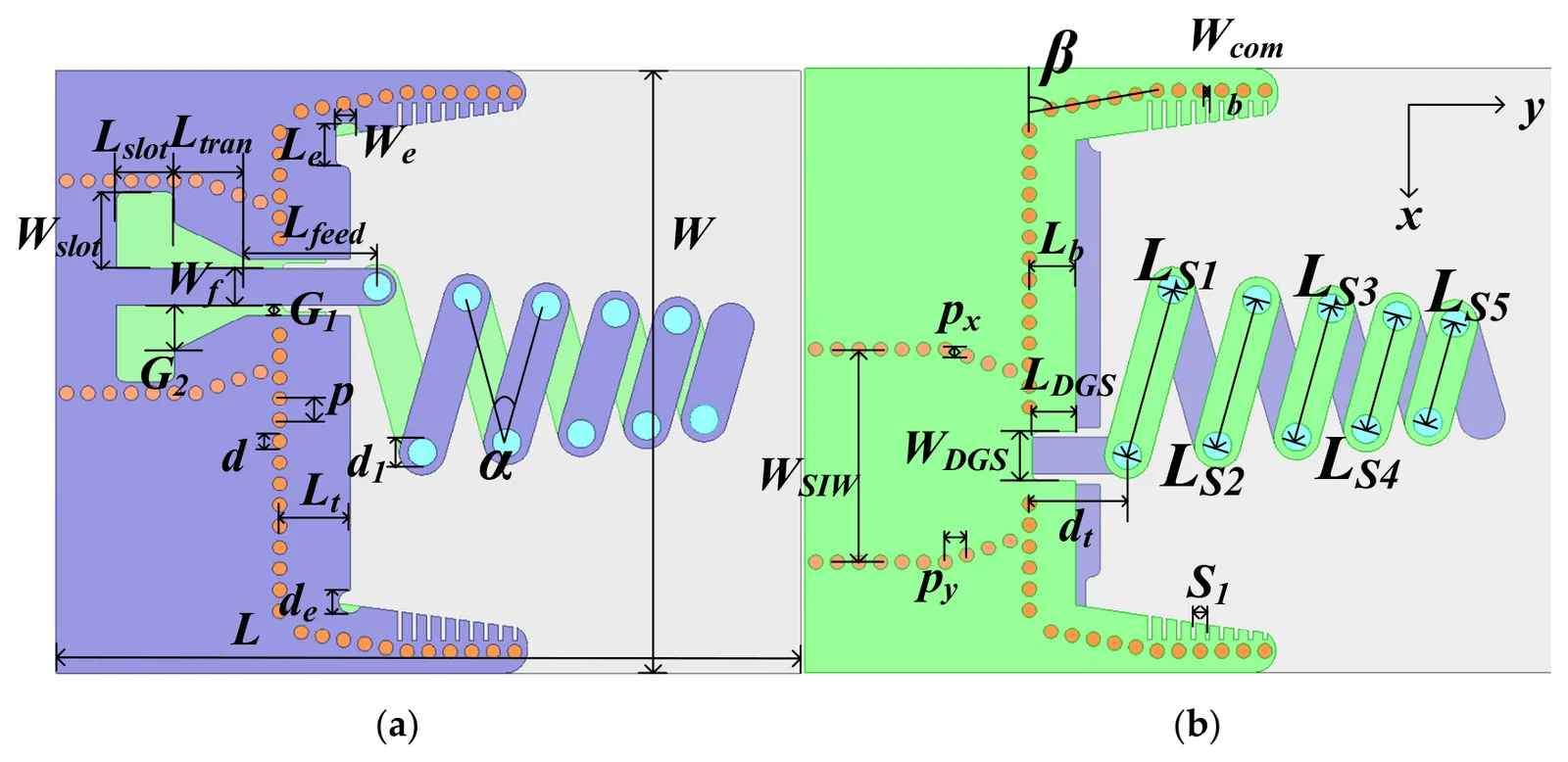
Configuration of the SIW-fed PHA. (**a**) Top side. (**b**) Back side.

The structure of the antenna unit is shown in Figure 1. The radiation structure of the antenna is composed of two layers of metal strips with progressively decreasing lengths interconnected by metallized through holes, and the input is realized by the transition from SIW to grounded coplanar waveguide (GCPW). Compared with the coaxial feeding mode [16], the proposed feeding structure is easier to connect to other devices. The length of the metal strip decreases according to the following equation:

(1) $$rate=\frac{L_{S\left(i+1\right)}}{L_{Si}},i=1,2,3,4$$

The key parameters that determine the operating frequency of the antenna are similar to those of the traditional helical antenna. When the length of the first helix is close to a wavelength, the antenna works in the end-fire radiation mode at the center frequency.

The metal strip and the ground metal behind the antenna together produce a horizontal surface current with a 90° phase difference from the vertical current generated by the metallized through hole [16,24,25,26]. Etching defected ground structure (DGS) and patterns on the ground metal can improve the antenna’s return loss and AR.

**Figure 2:**
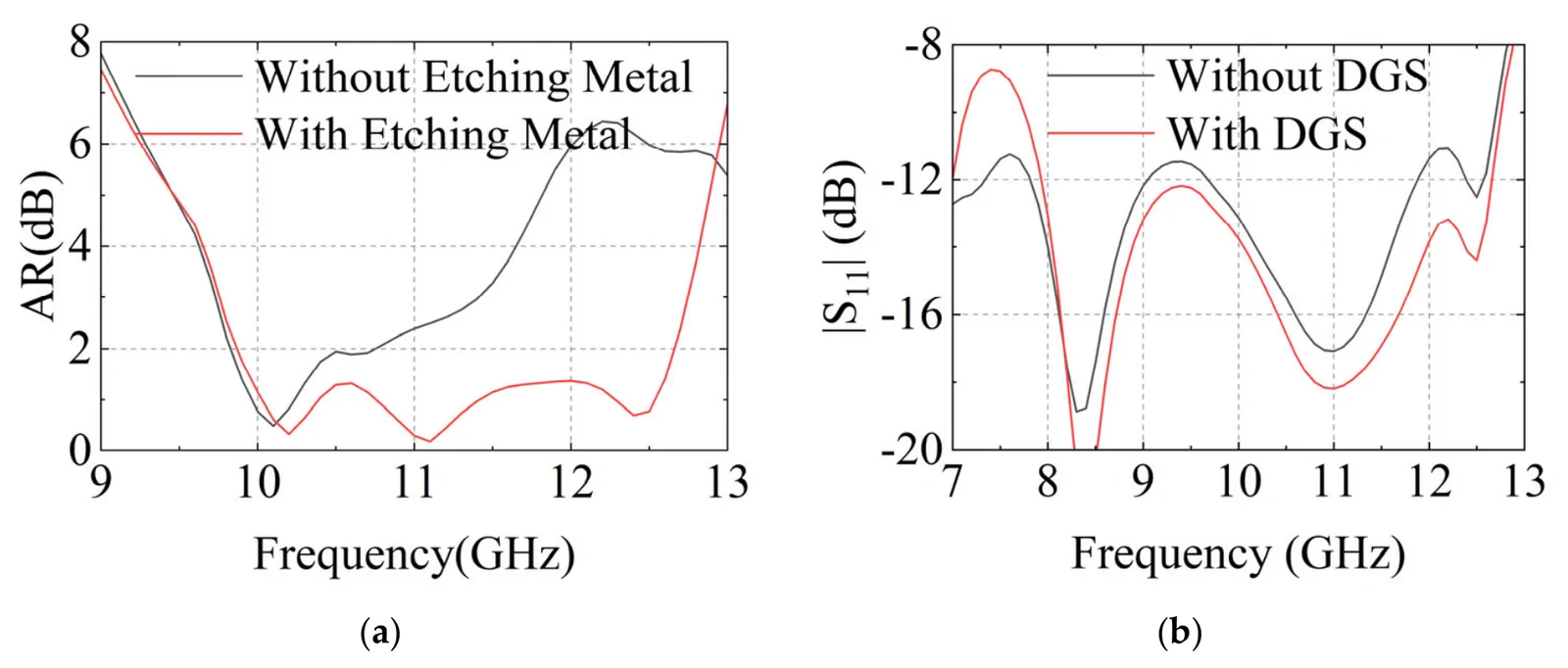
The influence of etched metal on antenna performance. (**a**) Effect of etched metal on AR. (**b**) Effect of DGS on antenna |S_11_|.

Figure 2 is a comparison diagram of the influence of whether the ground metal is etched or not on antenna performance. It can be seen that the axial ratio characteristics of the antenna can be significantly improved by etching the pattern on the ground metal, and the introduction of DGS can improve the impedance matching of the antenna while keeping other parameters unchanged.

**Figure 3:**
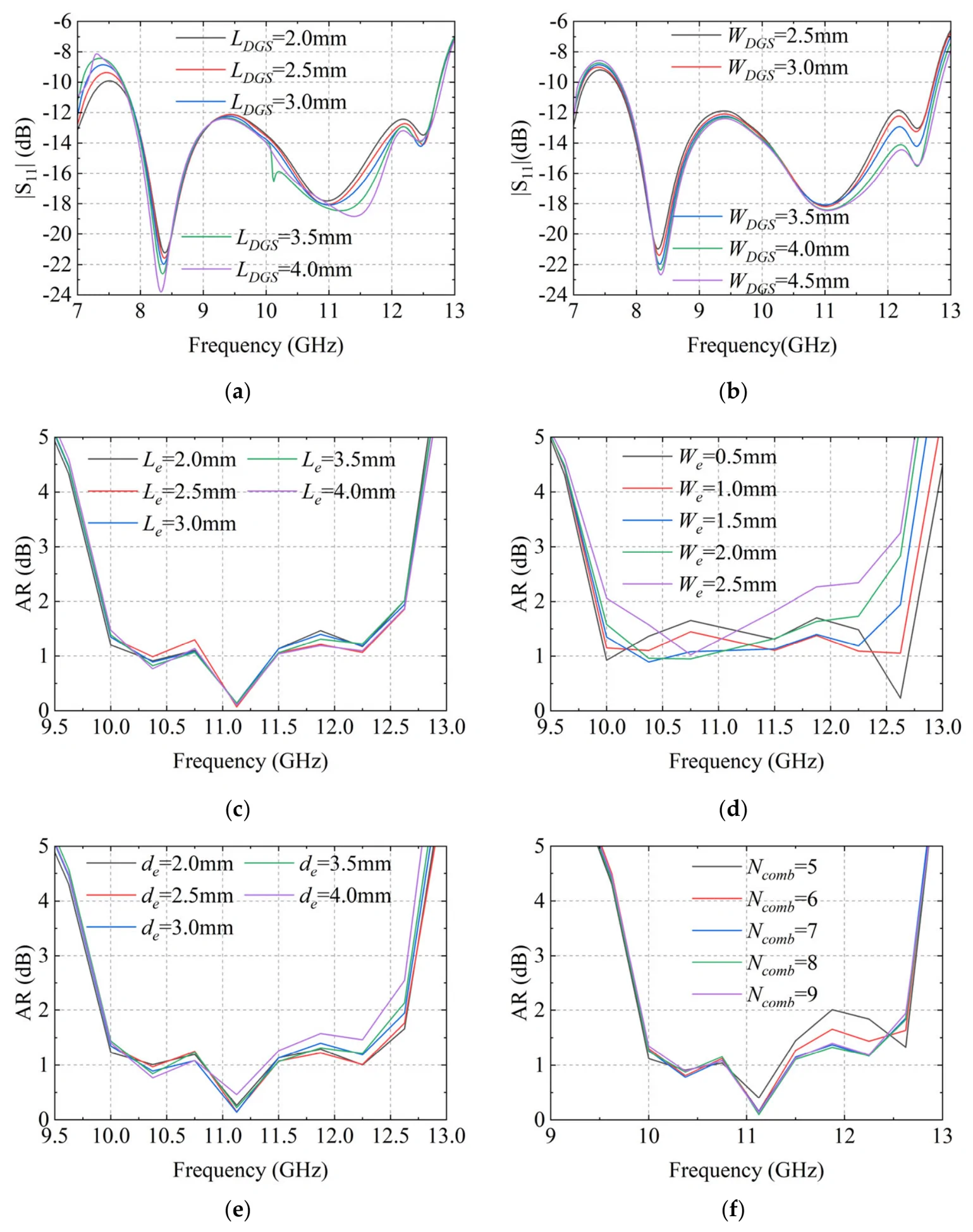
Effect of DGS and etched metal on antenna performance. (**a**) *L_DGS_* on |S_11_|; (**b**) *W_DGS_* on |S_11_|; (**c**) *L_e_* on AR; (**d**) *W_e_* on AR; (**e**) *d_e_* on AR; (**f**) *N_comb_* on AR.

A parametric analysis was carried out to evaluate the effects of the DGS and the three etched metal structures on antenna performance. During the analysis, only one geometric parameter was varied at a time, while all other parameters were kept constant. The influence of the dimensions of the DGS, square, circular, and comb-shaped etched metal structures on the antenna characteristics is presented in Figure 3.

As shown in Figure 3a,b, the *L_DGS_* and *W_DGS_* of the DGS mainly affect the impedance-matching performance of the antenna in the high-frequency band. Increasing either *L_DGS_* or *W_DGS_* leads to a lower |S_11_| level at higher frequencies, thereby improving the impedance matching in this frequency range.

The effects of the dimensions of the square etched metal structure on the antenna performance are shown in Figure 3c,d. The *L_e_* has a limited effect on the axial ratio (AR), whereas the *W_e_* has a more significant influence on the AR in the high-frequency band. As *W_e_* increases, the high-frequency AR initially decreases, indicating an improvement in the circular-polarization performance. When *W_e_* reaches 1.5 mm, the AR attains a relatively low value. However, a further increase in *W_e_* causes the high-frequency AR to increase.

The effect of the circular etched metal structure is shown in Figure 3e. With an increase in its diameter *d_e_*, the AR in the high-frequency band increases slightly, indicating a slight degradation in the circular-polarization performance. The influence of the *N_comb_* of the comb-shaped etched metal structure on the antenna AR is illustrated in Figure 3f. Increasing *N_comb_* generally reduces the AR and improves the circular-polarization performance. However, when *N_comb_* = 7, the AR becomes relatively insensitive to a further increase in *N_comb_*.

### 2.2. 1 × 4 PHA Array Analysis

The PHA described in the previous section has a wide lateral dimension. When the array is designed, its transverse size will lead to large array spacing, resulting in grating lobes that are difficult to eliminate. In order to avoid generating grating lobes, the helical body can be placed on the same dielectric substrate according to a certain array distance, and then the appropriate ground metal can be designed to optimize the performance of the antenna array. The appropriate array spacing is selected by *d_array_* in the following range [27]; *d*_array_ is the spacing between antenna units.

(2) $$\frac{1}{2}\lambda \leq d_{array}\leq \frac{3}{4}\lambda$$

**Figure 4:**
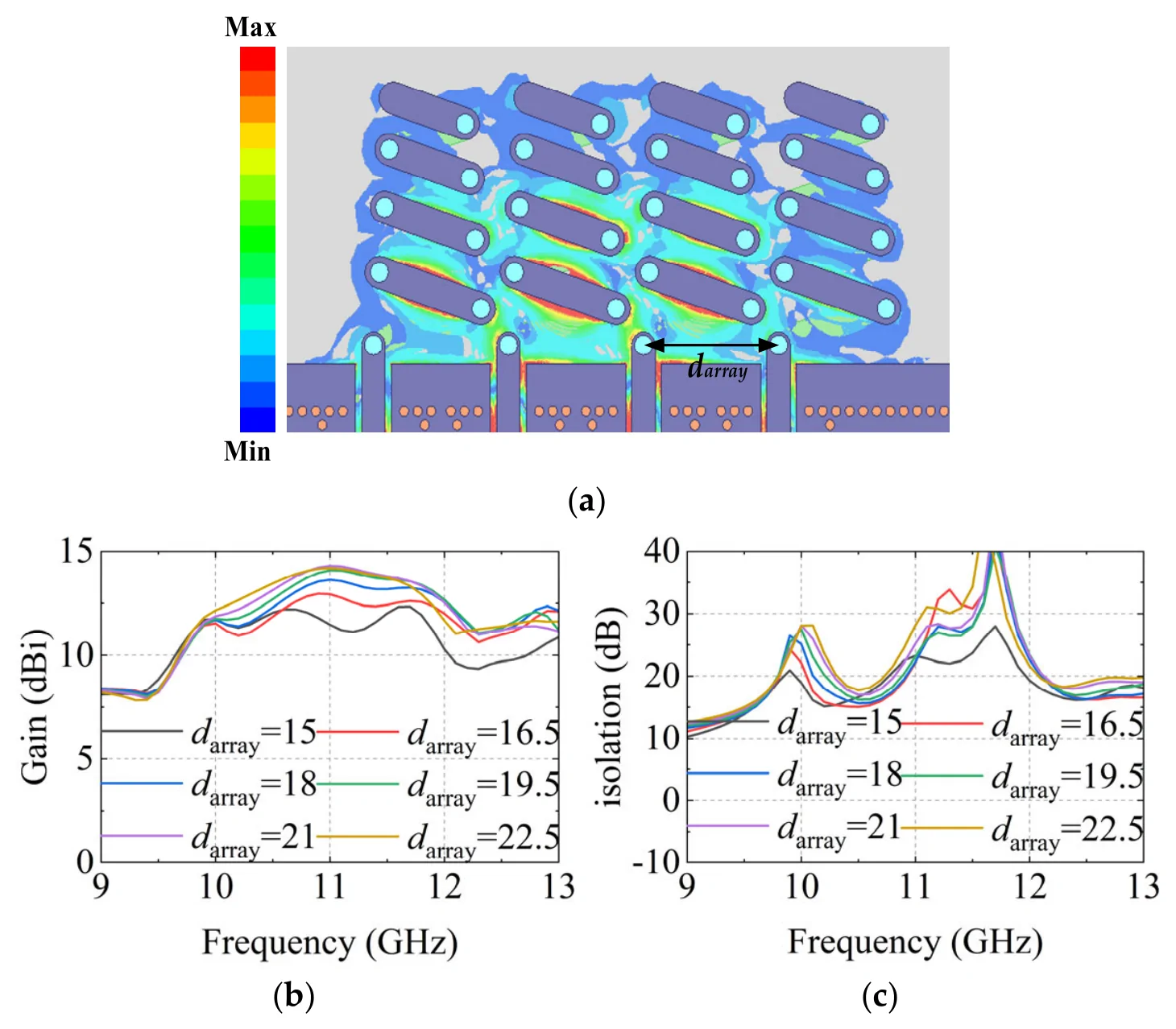
Analysis diagram of 1 × 4 PHA array. (**a**) Electric field distribution of 1 × 4 PHA array at 11 GHz. (**b**) The effect of array spacing on the gain of the antenna array. (**c**) Effect of array spacing on isolation between antenna elements. (Unit: mm).

However, in addition to the interaction between the array elements, the metal strips between the array elements also produce coupling effects. Figure 4a shows the electric field distribution on the antenna array when *d_array_* = 15 mm. As can be seen from Figure 4a, there is a strong energy distribution between the first and second ring of the antenna array metal strip and the antenna feeding line. It can be seen from Figure 4b that when *d*_array_ = 15 mm, the gain of the antenna array at 11 GHz is the lowest. With the increase in *d*_array_, the gain of the antenna array increases significantly. When *d*_array_ =19.5 mm, the gain of the antenna array is less affected by *d*_array_. It can be seen that the coupling of metal strips will affect the performance of the antenna array, and increasing *d*_array_ will increase the spacing of adjacent metal strips, reducing the impact of coupling on the radiation performance of the antenna array. The final horizontal array spacing used in this design is *d*_array_ =19.5 mm.

### 2.3. SIW Twist Transition Analysis

**Figure 5:**
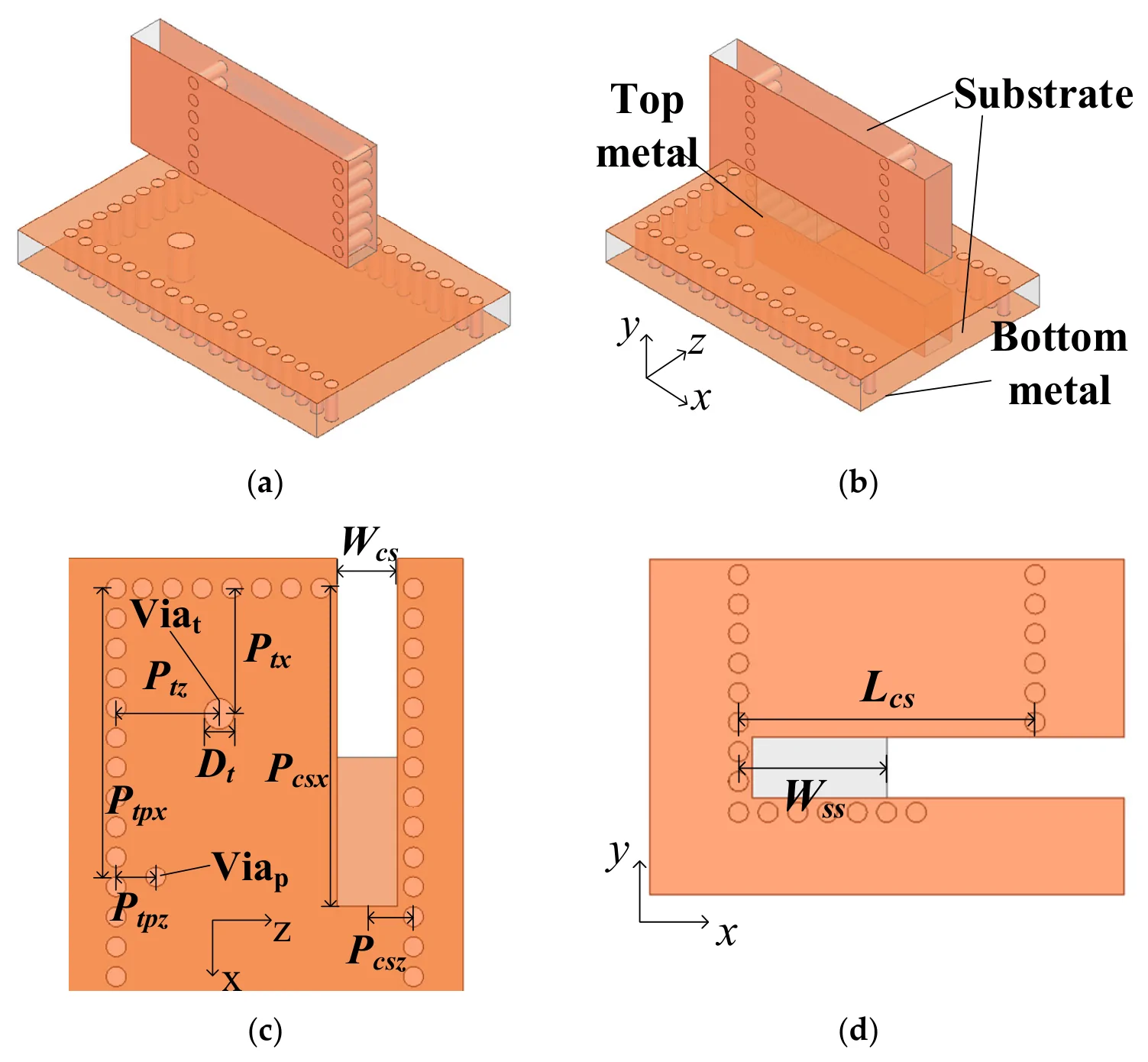
Configuration of the SIW twist. (**a**) The overall structure of SIW twist without occlusal structure; (**b**) with occlusal structure; (**c**) XOZ-plane SIW; and (**d**) XOY-plane SIW.

**Figure 6:**
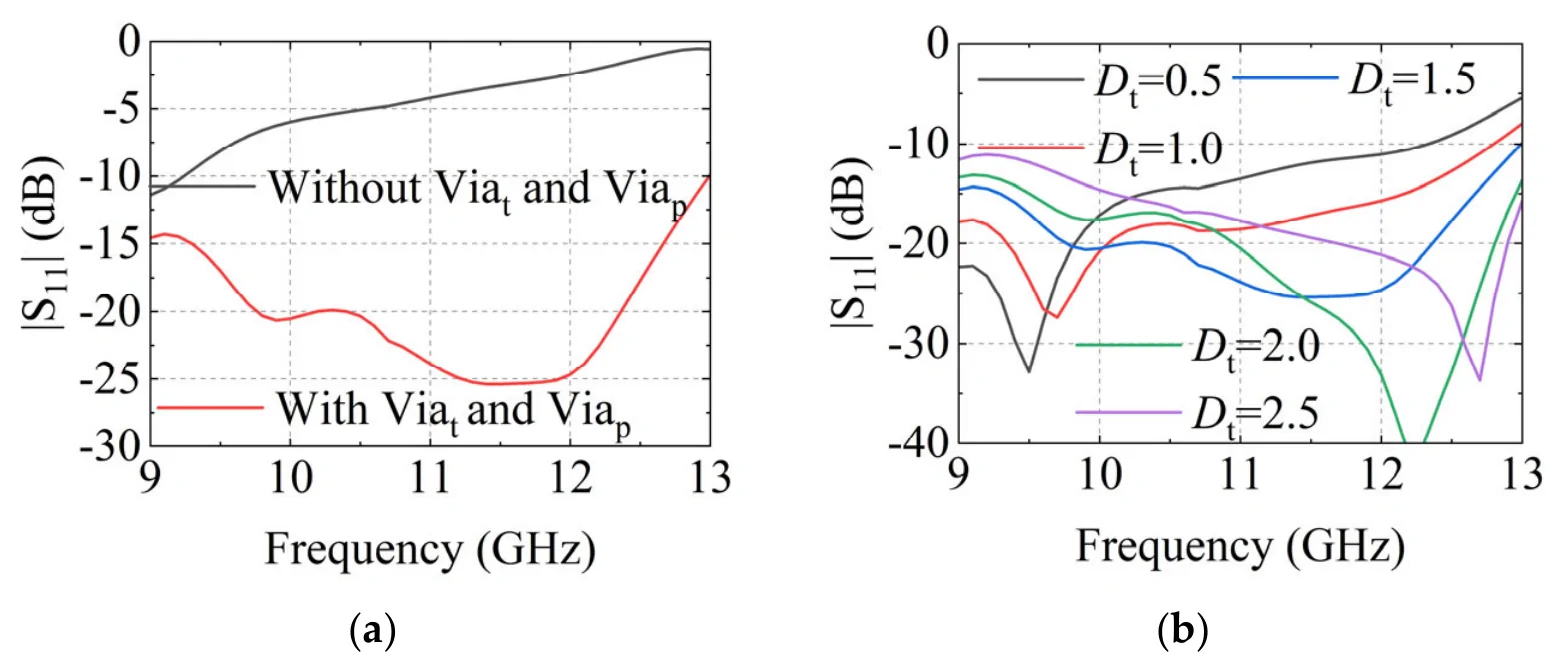

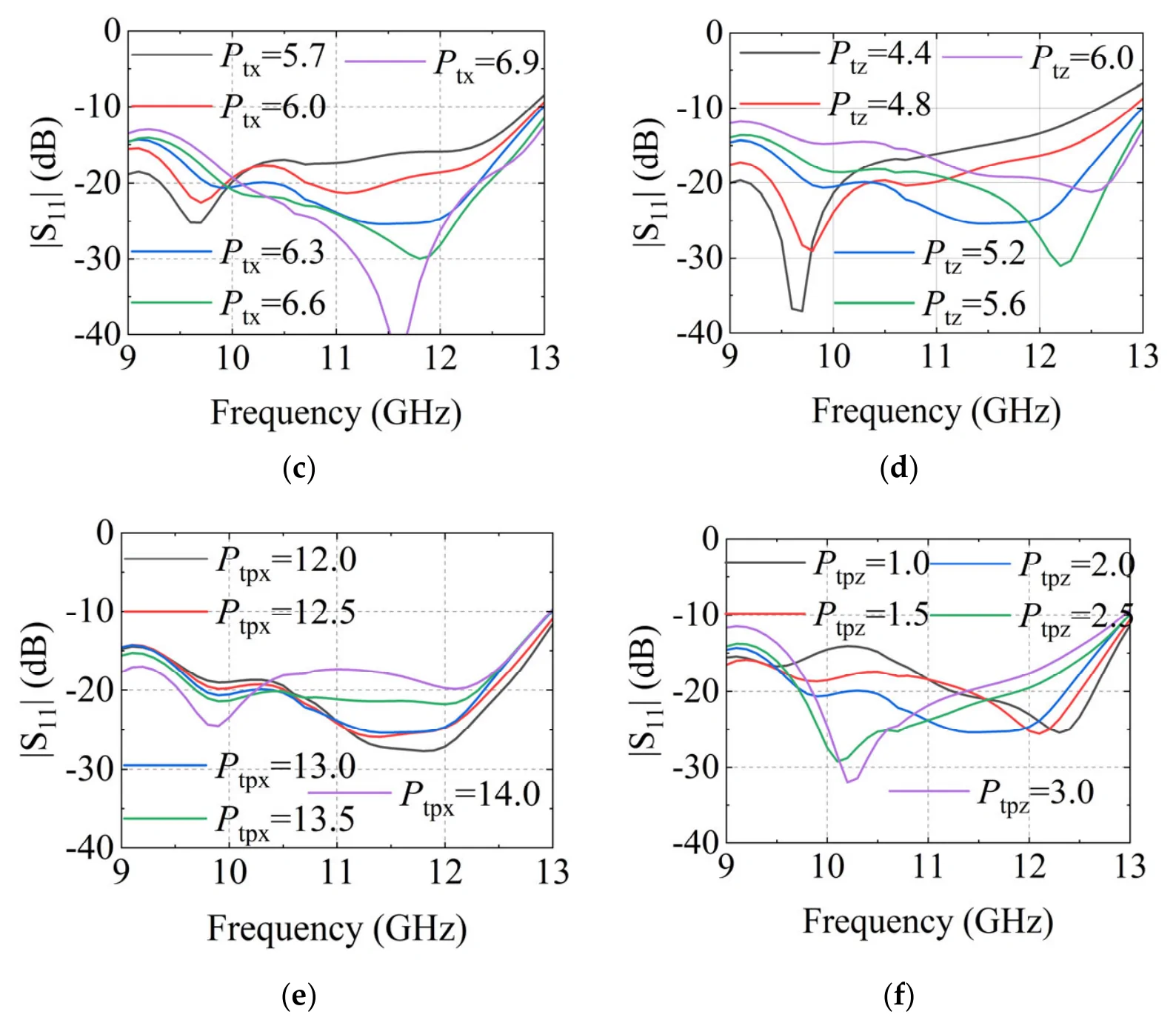
Influence of *Via_t_* and *Via_p_* on the return loss of the flecked waveguide. (**a**) Existence or not of *Via_t_* and *Via_p_*. (**b**) The diameter of *Via_t_*. (**c**) X-axis relative position of *Via_t_*. (**d**) Z-axis relative position of *Via_t_*. (**e**) X-axis relative position of *Via_p_*. (**f**) Z-axis relative position.

The structure of the SIW twist transition is shown in Figure 5. The twist transition consists of two SIWs that are vertically integrated into each other, transforming the horizontal TE_10_ mode into a vertical TE_10_ mode. This transition enables the transfer of energy from the horizontal direction to the vertical direction [28]. The twist waveguide transition can be regarded as a three-dimensional SIW corner structure. In this structure, two output ports perpendicular to two matching plated via holes are introduced into the corner of the structure, Via_t_ and Via_p_. Their effects are similar to those of planar SIW corners in which perpendicular plated via holes are introduced into SIW corners to compensate for the discontinuity of 90° corners and reduce the reflections caused by 90° corners. It can be seen that the introduction of two through holes greatly improved the return loss compared to without them. The main parameters are listed in Figure 5c,d. The effects of introducing Viat and Viap or not on return loss are shown in Figure 6a.

The positions and diameters of both through holes affect the return loss of the twist. In order to enable energy to be transmitted from the XOZ plane to the XOY plane, the top-layer metal on the SIW of the XOZ plane introduces a rectangular slot CS, whose width *W*_cs_ is consistent with the thickness of the substrate and whose length *L*_cs_ is consistent with *W_SIW_* in another measurement of matching plated via holes. Its position should be close to the edge of the SIW of the XOZ plane. With other parameters unchanged, the effect of the position and size of two through holes on the performance of the SIW twister is studied, and the results are shown in Figure 6. For *Via_t_*, increasing its diameter *D_t_* and its position *P_tx_* and *P*_ty_ along the x and z axes will improve the return loss at high frequencies but will worsen the return loss at low frequencies. However, for *D_t_* and *P_tz_*, after *d_t_* increases to 2.0 mm and *P_tz_* increases to 2.5 mm, the return loss at high frequency will become worse. For *Via_p_*, increasing *P_tpx_* in the x-axis direction and *P_tpy_* in the y-axis direction can improve the return loss at high frequency but worsen the return loss at low frequency. Based on the above research and comprehensive consideration, the design adopts the following parameters: *D_t_* = 1.5 mm, *P_tx_* = 6.3 mm, *P_tz_* = 5.2 mm, *P_tpx_* = 13 mm, *P_tpz_* = 2 mm, *P_csz_* = 2.34 mm, *L_cs_* = 14.3 mm, *W_cs_* = 3.01 mm, and Wss = 7.5 mm.

The basic structure of the SIW twist is shown in Figure 5a, which is placed vertically with the SIW of the XOY plane only after the top metal of the SIW of the XOZ plane is slotted, and the slotted size is equal to the size of the transverse section of the SIW. This method will cause dislocation in assembly, resulting in gaps at the joints that lead to energy leakage, affecting performance. In order to avoid the above situation, substrate slotting was performed at the joint of the two SIWs, as shown in Figure 5c,d. The substrate is hollowed out at the joint from the edge of the substrate to the center of the SIW in the XOY plane so that the two SIWs retain the length of the *W_SIW_*/2 dielectric substrate and retain the bottom metal of the SIW in the XOZ plane, where *W_SIW_* is the width of the SIW. In order to ensure that the energy can be better coupled to the vertical plane and simultaneously avoid actual machining errors and the influence of assembly, the width of the substrate slot *W_cs_* = 3.07 mm is slightly larger than the thickness of the substrate. Thus, the occlusal structure is formed to enhance the contact tightness and reduce the assembly difficulty.

The SIW twist design procedure is summarized as follows:

(1) Basic structure design: The top metal slot is placed at the metal wall near the SIW edge of the XOZ plane; the length *L_CS_* and width *W_CS_* of the slot are close to *W_SIW_* and the thickness of the substrate.

(2) Optimize performance: Optimize *Via_t_* and *Via_p_* positions and sizes to get the best return loss.

(3) Design the occlusal structure: Refer to the form in Figure 5c,d and the above discussion, design an occlusal structure that is easy to assemble, and adjust the position and size of *Via_t_* and *Via_p_* to obtain the best performance.

(4) Processing and assembly: After the two pieces of SIW are orthogonally assembled, weld at the joint to enhance the tightness of the structure.

### 2.4. 4 × 4 PHA Array Design

**Figure 7:**
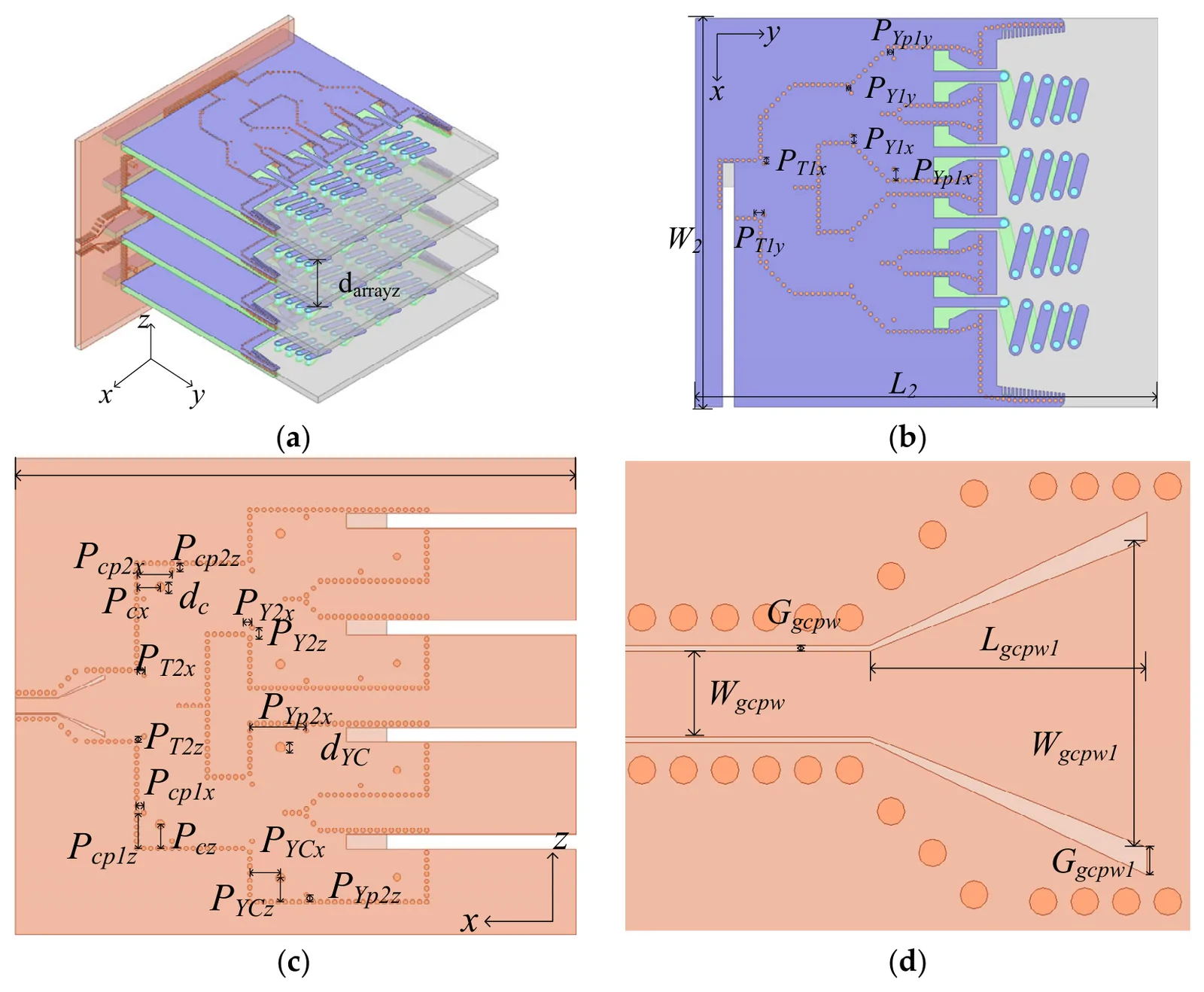
Configuration of the 4 × 4 PHA array. (**a**) Overall structure; (**b**) 1 × 4 PHA array structure; and (**c**) XOZ plane SIW power divider; (**d**) SIW-to-GCPW transition structure.

The antenna array is shown in Figure 7a. The array takes a 1 × 4 PHA array as the basic unit and is constructed as a 4 × 4 array with an array spacing *d_arrayz_* = 22.5 mm along the z-axis. The 1 × 4 subarray and the SIW power splitter are cascaded on the same substrate and then vertically connected to the YOZ-plane SIW power splitter.

Details of the XOZ plane SIW power divider are shown in Figure 7c. The power splitter is in parallel to maintain the phase consistency of the output port, and the output port is converted from the SIW-GCPW transition structure [29] to GCPW for testing. The output ports and the input end of the 1 × 4 subarray are connected vertically by the SIW twist described in the previous section. In order to suppress the back lobe, the length of the power divider is extended so that the top metal forms a square ground plane similar to that of a traditional spiral antenna.

## 3. Experimental Results

**Figure 8:**
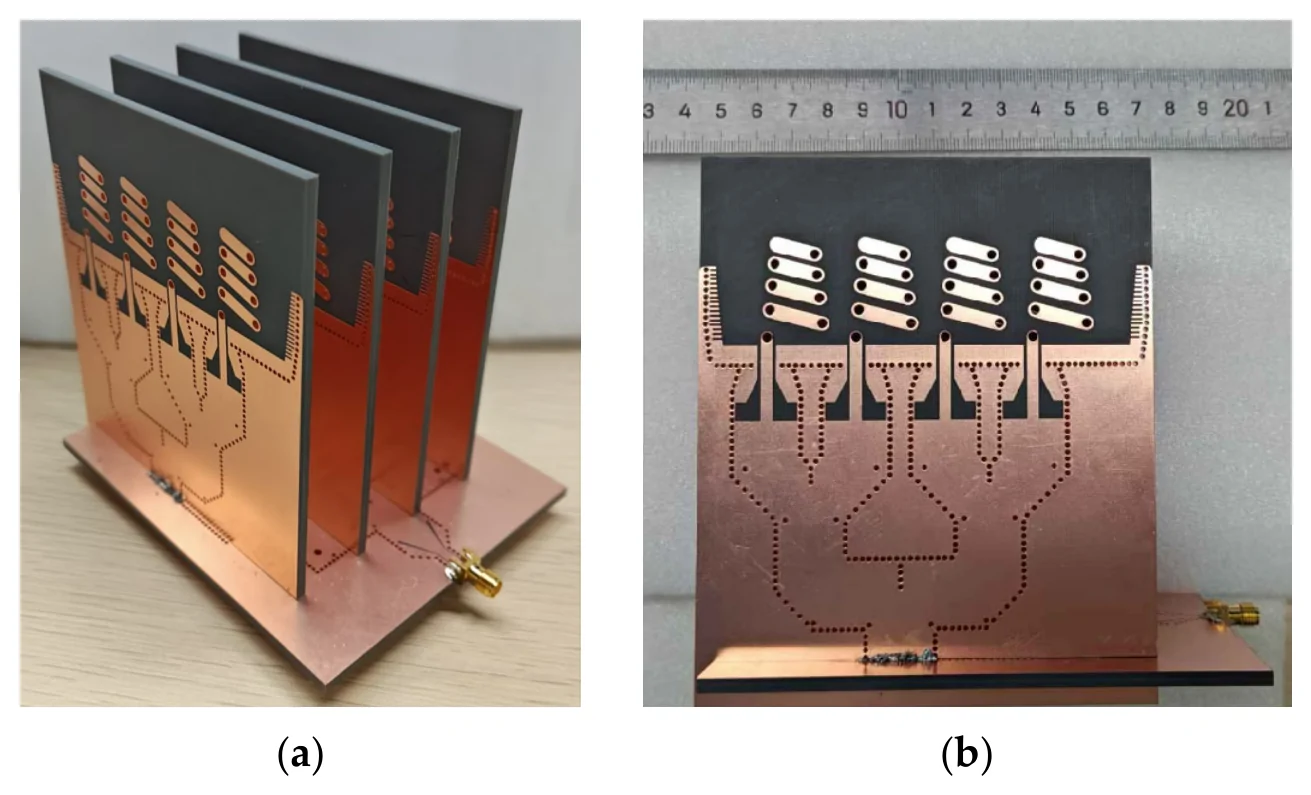
Prototype of the proposed 4 × 4 PHA array. (**a**) Full view; (**b**) side view.

**Figure 9:**
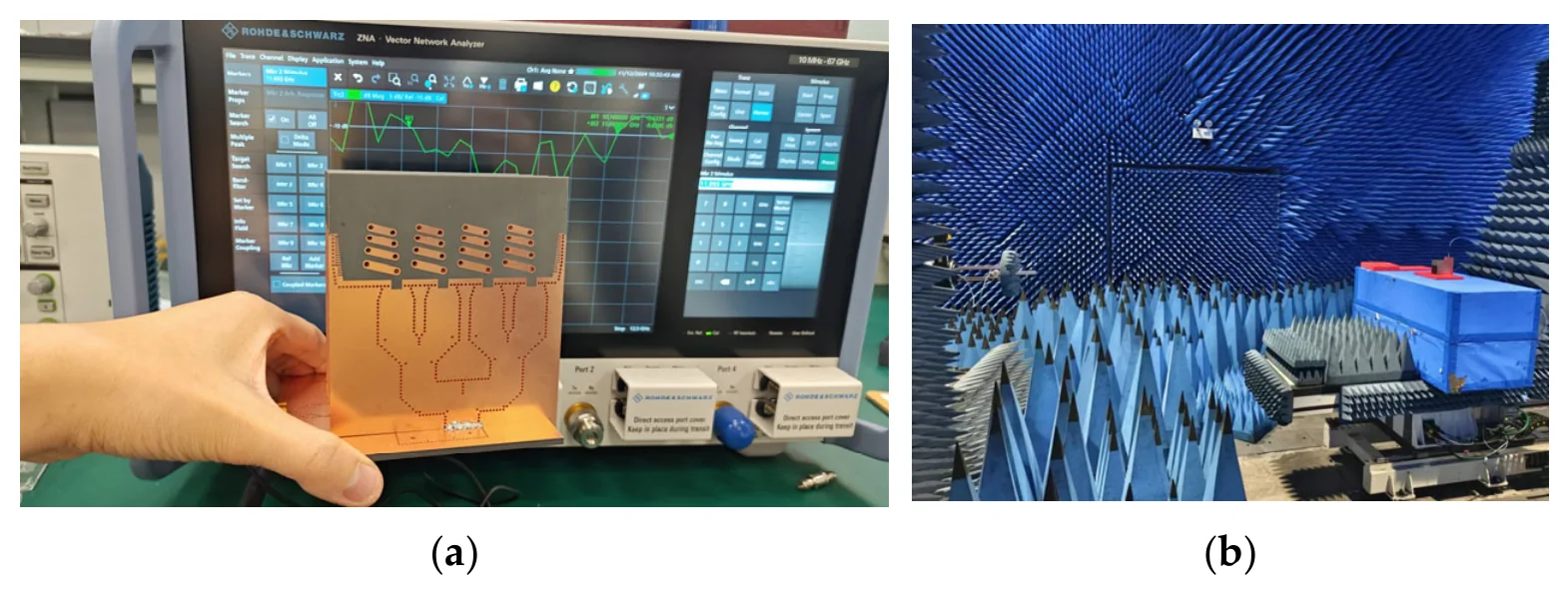
4 × 4 PHA array measurement environment. (**a**) Measurement by vector network analyzer; (**b**) radiation pattern measurement in the microwave anechoic chamber.

A prototype is fabricated to characterize the performance of the proposed 4 × 4 PHA array, as shown in Figure 8, and the detailed parameters of the 4 × 4 PHA array are shown in Table 1. The S-parameter is measured by the Rohde & Schwarz ZNA67 vector network analyzer, and the radiation pattern, axial ratio and actual gain of the antenna array are measured in the microwave anechoic chamber. Pictures of the test process are shown in Figure 9.

**Figure 10:**
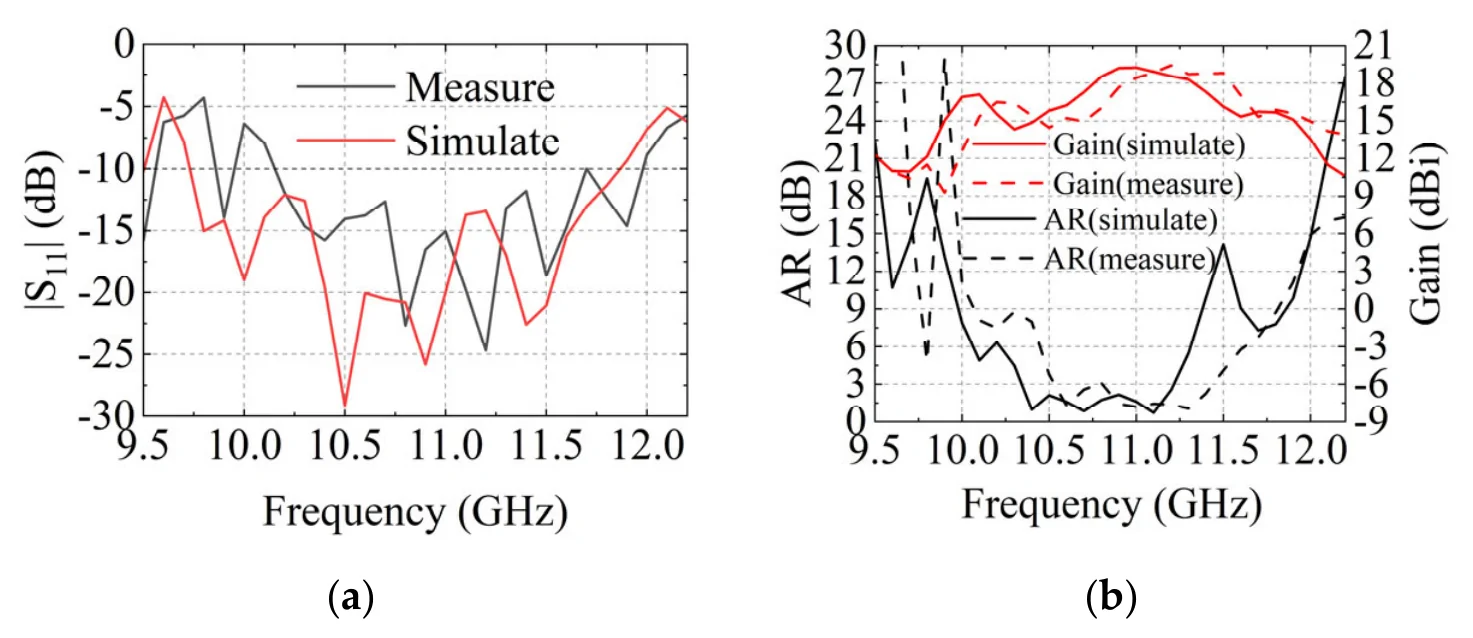
Simulated and measured results of the 4 × 4 PHA array. (**a**) |S_11_|; (**b**) AR and gain.

As shown in Figure 10, IBW simulation results of the antenna array range from 9.73 to 11.87 GHz by 19.45%, and measured results range from 10.16 to 11.99 GHz by 16.64%. ARBW simulation results range from 10.34 to 11.18 GHz at 7.64%, and measured results range from 10.53 to 11.47 GHz at 8.55%. The simulation and measurement maximum gain of 11 GHz is 19.25 dBi and 18.44 dBi, respectively. Compared with the simulation results, the center frequency offsets, |S_11_|, AR and gain differences are mainly due to the frequency offset caused by the assembly gap caused by the slot deformation of the substrate caused by machining errors and the size deviation of the metal strip.

**Figure 11:**
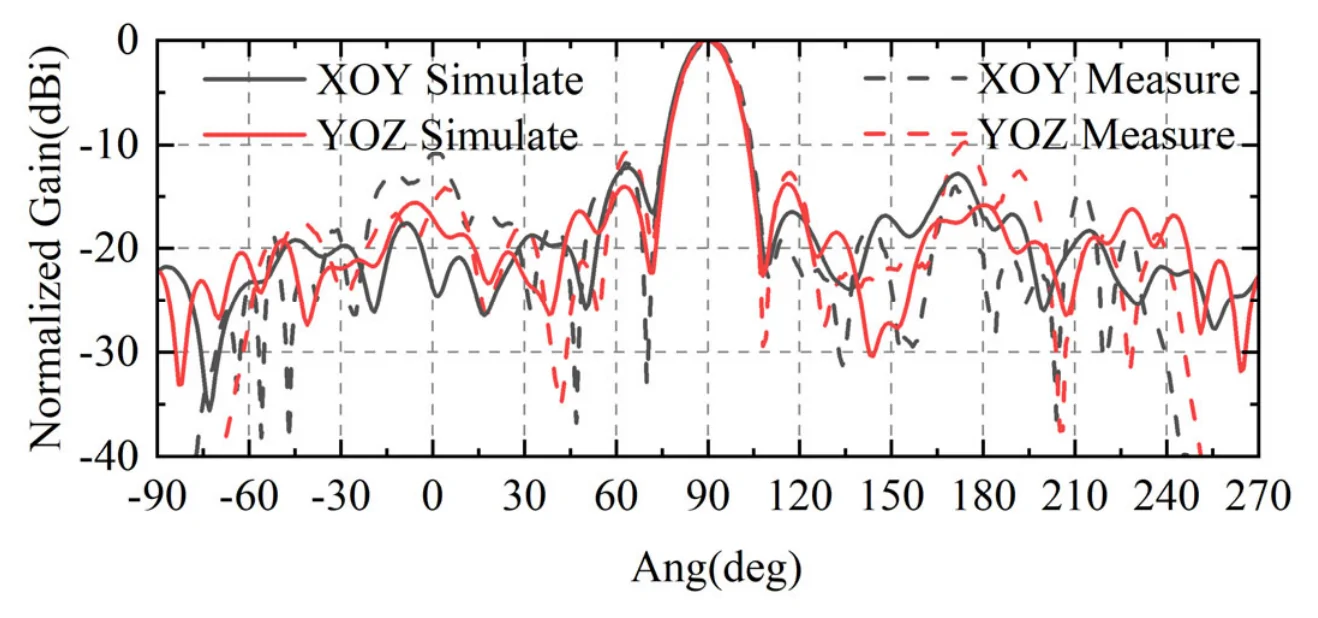
Simulated and measured radiation pattern of the 4 × 4 PHA array at 11 GHz.

The XOY and YOZ plane simulations of the antenna array at 11 GHz are highly consistent with the measured normalized pattern, as shown in Figure 11. The array spacing is chosen to reduce mutual coupling, resulting in a sidelobe level of 12.68 dB, while the front-to-back ratio is raised to 22.4 dB by a SIW power splitter perpendicular to the radiation direction. The results show that the method proposed in this paper can significantly enhance array directivity and achieve a high front-to-back ratio while ensuring low mutual coupling, and the feed network composed of SIW can also reduce energy transmission loss.

As shown in Table 2, the proposed array exhibits a competitive gain performance compared with previously reported planar circularly polarized antennas and arrays. In particular, the measured peak gain of 18.44 dBi is approximately 3.2 dB higher than that of the 4 × 4 LTCC helical antenna array in [14]. Compared with the planar helical antenna in [16] and the SIW-fed double-helix antenna in [15], the proposed three-dimensional array architecture provides a substantial gain enhancement while retaining the end-fire circularly polarized radiation characteristics. Moreover, compared with the 4 × 4 planar CP arrays in [18,19], whose reported peak gains are 18.28 and 17.85 dBi, respectively, the proposed array achieves a slightly higher peak gain of 18.44 dBi.

More importantly, unlike conventional planar CP arrays, the proposed design addresses the array-integration problem inherent in planar end-fire helical antennas. By introducing a three-dimensional SIW feeding network and a 90° SIW twist transition, four 1 × 4 end-fire PHA subarrays can be orthogonally interconnected to form a 4 × 4 array. Therefore, the main advantage of the proposed antenna lies in the combination of high gain, circular polarization, end-fire radiation, low-loss SIW feeding, and PCB-compatible three-dimensional array integration.

## 4. Conclusions

In this work, a three-dimensional SIW-fed 4 × 4 planar helical antenna array has been proposed and experimentally demonstrated. The main contribution of this work is a three-dimensional array integration architecture that enables four 1 × 4 end-fire PHA subarrays to be combined into a 4 × 4 array through an orthogonal SIW feeding network. To realize this architecture, a 90° SIW twist transition incorporating matching metallized vias and an interlocking structure is developed, which enables efficient TE_10_-mode transformation while facilitating practical PCB assembly. In addition, the element spacing and feeding structure are optimized to reduce mutual coupling and suppress backward radiation. The fabricated prototype operating around 11 GHz achieves a measured gain of 18.44 dBi, an impedance bandwidth of 16.64%, an axial ratio bandwidth of 8.55%, and a front-to-back ratio of 22.4 dB. These results demonstrate that the proposed architecture provides a practical approach for realizing compact, high-gain, circularly polarized end-fire antenna arrays using standard PCB and SIW technologies. The proposed antenna is potentially suitable for X-band satellite communication, radar, and other long-range wireless systems requiring high gain and circular polarization.

## Figures and Tables

**Table 1:** Dimensions of the proposed 4 × 4 PHA array.

Parameter	Value (mm)	Parameter	Value (mm)	Parameter	Value (mm)
*W*_2_	100.4	*G*_2_	3.2	*P*_*cp*2*x*_	7.5
*L*_2_	119.82	*L_feed_*	9.16	*P*_*cp*2*z*_	1.5
*H*	3	*L_tran_*	5	*P*_*Y*2*x*_	0.5
*W_SIW_*	15	*L_slot_*	4	*P*_*Y*2*z*_	1.5
*d*	1	*W_slot_*	5.4	*d_YC_*	2
*d*_1_	2	*p_x_*	0.5	*P_YCx_*	6.5
*p*	1.5	*p_y_*	1.5	*P_YCz_*	5
*L*_*S*1_	2.3	*d_array_*	19.5	*P*_*Yp*2*x*_	12
*rate*	0.86	*d_arrayz_*	22.5	*P*_*Yp*2*z*_	1.5
*α*	28°	*P*_*T*1*x*_	1.6	*P_tpx_*	13
*W_a_*	3.2	*P*_*T*1*y*_	0.9	*P_tpz_*	2
*L_t_*	4.3	*P*_*Y*1*x*_	2	*D_t_*	1.5
*L_b_*	2.6	*P*_*Y*1*y*_	1	*P_tx_*	6.3
*d_t_*	6.15	*P*_*YP*1*x*_	3	*P_tz_*	5.2
*L*_1_	5.5	*P*_*YP*1*y*_	1.5	*W_CS_*	3.07
*L*_2_	21	*W*_3_	100	*L_CS_*	14.3
*L*_3_	3.5	*L*_3_	119	*P_CSX_*	17
*β*	82°	*P*_*T*2*x*_	1.6	*P_CSZ_*	2.34
*W_comb_*	0.5	*P*_*T*2*z*_	1.1	*W_SS_*	7.5
*S_comb_*	1	*d_c_*	2	*W_gcpw_*	3.1
*L_DGS_*	4	*P_cx_*	5	*G_gcpw_*	0.2
*W_DGS_*	4.4	*P_cz_*	5	*W*_*gcpw*1_	11.1
*W_f_*	2.6	*P*_*cp*1*x*_	1.5	*G*_*gcpw*1_	1
*G*_1_	0.7	*P*_*cp*1*z*_	7.5	*L*_*gcpw*1_	10

**Table 2:** Comparison of the proposed antenna with previous work.

Ref.	Year	Antenna Type	Array	Feeding/Fabrication	IBW (%)	ARBW (%)	Peak Gain (dBi)
[14]	2012	Planar helical	4 × 4	GCPW/stripline, LTCC	22	20	15.2
[15]	2022	Double-helix	1 × 1	SIW, PCB	28.6	—	7.8
[16]	2015	Planar helical	1 × 1	Coaxial–GCPW, PCB	54	34	8.0
[17]	2014	Helical + SIH	2 × 2	LTCC	16.2	22.2	11.8
[18]	2020	CP dipole	4 × 4	Planar integrated PCB	21.83	5.9	18.28
[19]	2021	CP dipole + parasitic patches	4 × 4	SIW, PCB	27.7	28.5	17.85
This work	—	Planar helical	4 × 4	3-D SIW, PCB	16.64	8.55	18.44

## Data Availability

The original contributions presented in this study are included in the article. Further inquiries can be directed to the corresponding author.
